# Effects of single femoral nerve block and continuous femoral nerve block on perioperative analgesia and muscle strength in elderly patients undergoing total knee arthroplasty, a randomized clinical trial

**DOI:** 10.3389/fsurg.2025.1403280

**Published:** 2025-06-16

**Authors:** Yan Tao, Nan Cai, Juxia Zhang, Yan Zhou, Pengfei Liu

**Affiliations:** ^1^ Department of Pain, Beijing Jishuitan Hospital, Capital Medical University, Beijing, China; ^2^ Department of Anesthesiology, Beijing Jishuitan Hospital, Capital Medical University, Beijing, China; ^3^ Department of Anesthesiology, Beijing Shijitan Hospital, Capital Medical University, Beijing, China

**Keywords:** ropivacaine, femoral nerve block, perioperative analgesia, total knee arthroplasty, the elderly

## Abstract

**Objective:**

To investigate the effect of single femoral nerve block (SFNB) with 0.2% ropivacaine 50 ml on postoperative pain and muscle strength in elderly patients undergoing knee replacement.

**Methods:**

Ninety-four patients scheduled for primary total knee arthroplasty (TKA) were randomized into two groups. The patients in the SFNB group received SFNB with 50 ml 0.2% ropivacaine (*n* = 48), while the patients in the continuous femoral nerve block (CFNB) group (*n* = 46) received CFNB with an initial load of 20 ml 0.5% ropivacaine and a continuous injection of 0.2% ropivacaine at a rate of 5 ml/h. After the surgery, all patients were administered patient-controlled intravenous analgesia. The primary outcome was the visual analogue scale (VAS) score at 24 h postoperatively. The secondary outcomes included: (a) Pain scores at 2 h, 6 h, 12 h, 48 h, and 72 h after surgery, and the total dosage of celecoxib; (b) Muscle strength of the quadriceps at 2 h, 6 h, 12 h, 24 h, 48 h, and 72 h postoperatively; (c) Range of motion (ROM) at 24 h, 72 h, and 1 week after surgery; (d) American Knee Society knee score (AKS) at 1 week postoperatively; (e) Related complications (e.g., nausea and vomiting), and length of hospitalization; (f) General Comfort Questionnaire (GCQ) score at 72 h postoperatively.

**Results:**

(a) There were no statistically significant differences in VAS scores (*p* > 0.05) or the total dosage of celecoxib (*p* > 0.05) between the two groups at various time points; (b) The muscle strength scores in the SFNB group were higher than those in the CFNB group (*p* < 0.05) at 6 h, 12 h, and 24 h postoperatively; (c) Knee ROM in the SFNB group was better than in the CFNB group (*p* < 0.05); (d) There were no significant differences in adverse events between the two groups (*p* > 0.05); (e) The physiological and psychological scores on the GCQ in the SFNB group were higher than those in the CFNB group (*p* < 0.05).

**Conclusion:**

SFNB, with 0.2% ropivacaine 50 ml provides effective pain relief, and improves patient comfort after surgery, without increasing adverse effects. SFNB is a safe and convenient option for postoperative pain management following knee surgery.

## 1 Introduction

Total knee arthroplasty (TKA) is highly effective for treating arthritis, significantly improving knee function (1). Effective postoperative pain management is crucial for rapid recovery (2).

Continuous femoral nerve block (CFNB) is considered the “gold standard” for postoperative pain control after TKA, reducing opioid use (3, 4). However, it can delay quadriceps strength recovery and cause complications like catheter prolapse or increased discomfort in elderly patients. Thus, exploring a simpler analgesic protocol is clinically important.

Recently, nerve blocks using high-volume, low-concentration anesthetics have gained attention, as they reduce motor block while providing long-lasting analgesia (5, 6). This study aimed to compare the analgesic duration and effectiveness of single femoral nerve block (SFNB) with 0.2% ropivacaine 50 ml vs. CFNB with 0.2% ropivacaine through a randomized controlled trial, to improve pain management strategies.

## 2 Method

### 2.1 General information

This study was approved by the Medical Ethics Committee of Beijing Jishuitan Hospital and registered with the China Clinical Trial Center (number: ChiCTR2100047747). All patients and their families signed informed consent forms prior to the trial. A total of 100 patients (57 males and 43 females, aged 65–90 years) undergoing TKA from June 2021 to October 2022 were selected and randomly divided into two groups: the SFNB group and the CFNB group.

### 2.2 Subjects

**Inclusion criteria:**

(1) Age >65 years,
(2) ASA Grades (American Society of Anesthesiologists Grades) ≤Grade III,
(3) Undergoing knee replacement with spinal anesthesia, and the operation time was less than 3 h.

**Exclusion criteria:**

(1) Refusal to participate in the study,
(2) Patients with abnormal coagulation function or those recently taking anticoagulant/antiplatelet drugs,
(3) Patients with schizophrenia, epilepsy, Parkinson's disease, or myasthenia gravis,
(4) Inability to communicate due to coma, severe dementia, or speech disorder,
(5) Recent history of craniocerebral injury, neurosurgery, or spinal surgery,
(6) Patients with sick sinus syndrome, severe sinus bradycardia (heart rate <50 beats/min), or severe atrioventricular block without pacemaker implantation,
(7) Severe abnormal liver function (Child-Pugh grade C),
(8) Severe renal dysfunction (preoperative dialysis),
(9) ASA Grade IV and above,
(10) Patients with skin rupture, infection, vasculitis, or local surgical needs in the groin area.
(11) Patients with chronic pain (VAS score >6), including severe joints pain, low back pain, and tumor pain.

**Rejection criteria**:

(1) Failed spinal anesthesia,
(2) Patients who experienced severe hypotension, severe allergic reactions, or toxic reactions to local anesthetic drugs during surgery,
(3) Patients who developed severe delirium during or after surgery.

### 2.3 Anesthesia method

In the operating room, all patients underwent continuous monitoring of ECG, oxygen saturation, and blood pressure. A peripheral intravenous route was established before surgery, and 5 ml/kg/h of Ringer's lactate solution was infused intravenously. Oxygen was administered at 3–5 L/min via a mask. The radial artery was punctured and catheterized under local anesthesia with 1% lidocaine to monitor invasive arterial pressure. Subarachnoid block was routinely performed in both groups. This block was administered at the L3–4 lumbar space using 0.2% ropivacaine, 3.0 ml. The degree of sensory block (assessed by a temperature test) was evaluated by anesthesiologists not involved in the study, and the anesthesia plane was adjusted to the T10 level. Intraoperatively, systolic blood pressure (SBP) was maintained at no less than 80% of the baseline level. Atropine and norepinephrine were prepared to manage bradycardia (heart rate <50 bpm or below 80% of baseline) and hypotension (SBP <90 mmHg or below 80% of baseline SBP).

Management of adverse events during the operation: (1) Norepinephrine was continuously infused at an initial dose of 8–12 μg/min to prevent and treat hypotension (SBP <80% of baseline); (2) Ondansetron 8 mg was administered to prevent and treat nausea and vomiting; (3) Atropine 0.25 mg was used to prevent and treat bradycardia (heart rate <50 beats/min); (4) Patients experiencing allergic reactions were treated with 80 mg methylprednisolone. The internal environment was adjusted based on blood gas analysis results.

At the end of the procedure, all patients underwent ultrasound-guided femoral nerve block and were randomly divided into two groups: SFNB and CFNB.

Upon entering the room, patients were positioned supine for the ultrasound-guided sciatic nerve block. The ultrasound probe was placed horizontally, 2 cm below the inguinal ligament, with its long axis perpendicular to the longitudinal axis of the thigh. The femoral vein and femoral artery were clearly visualized, with the sciatic nerve arranged from the inside to the outside of the inguinal ligament.

A 20G venous catheter needle was inserted through the sartorius muscle to reach the femoral nerve, located on the surface of the iliopsoas muscle. A small amount of 0.9% sodium chloride solution was injected to observe the diffusion.

In the SFNB group, patients received 50 ml of 0.2% ropivacaine (Registration number: H20140763, AstraZeneca AB Sweden, 10 ml:100 mg) injected around the femoral nerve. In the CFNB group, patients were initially injected with 20 ml of 0.5% ropivacaine around the femoral nerve, followed by the placement and fixation of a catheter for postoperative self-controlled analgesia. The analgesic pump contained 250 ml of 0.2% ropivacaine, with a background dose of 5 ml, a bolus of 5 ml, and a lock-out time of 30 min.

At the end of the surgery, all patients were transferred to the surgical intensive care unit (SCIU). If postoperative pain exceeded 4 (VAS score >4), 200 mg of celecoxib was administered orally.

### 2.4 Observation indicators

#### 2.4.1 Primary outcome

The visual analogue scale (VAS) was used to assess pain (both at rest and during exercise) 24 h after surgery. The VAS score ranges from 0 to 10 points, with higher scores indicating more severe pain.

#### 2.4.2 Secondary outcome

① VAS (at rest and during exercise) was measured at 2 h, 6 h, 12 h, 48 h, and 72 h post-surgery, along with the dosage of remedial analgesic celecoxib.

② Quadriceps muscle strength was evaluated using the manual muscle test (MMT) at 2 h, 6 h, 12 h, 24 h, 48 h, and 72 h after surgery (7). The MMT score ranges from 0 to 5, where: 5 indicates normal resistance to gravity and external force, 4 indicates resistance to gravity and partial resistance, 3 indicates resistance to gravity but not to additional resistance, 2 indicates no resistance to gravity, but with full joint movement, 1 indicates muscle contraction without joint movement, 0 indicates complete paralysis with no muscle contraction.

③ Postoperative rehabilitation was assessed by measuring the range of knee joint motion (ROM) at 24 h, 72 h, and 1 week after surgery (8). The American Knee Society Knee Score (AKS) was used to evaluate functional status 1 week postoperatively. The AKS consists of two components: The knee score includes pain (50 points), ROM (25 points), and stability (25 points), with points deducted for knee flexion and extension contracture. The functional score evaluates walking ability (50 points) and stair climbing ability (50 points), with deductions made for functional impairments.

④ Complications occurring during hospitalization were recorded, including nausea, vomiting, headache, vertigo, fever, hypotension, bradycardia, infections (urinary system, surgical incision, lung, etc.), deep vein thrombosis of the lower limbs, urinary retention, pruritus, catheter prolapse, and the length of hospital stay.

⑤ The General Comfort Questionnaire (GCQ) was used to assess patient comfort 72 h after surgery (9). The scale includes 28 items across four dimensions: physiological, mental, social-cultural, and environmental. A Likert scale from 1 to 4 was used for scoring, with higher scores indicating greater comfort.

### 2.5 Randomization process and allocation concealment

All patients were assigned numbers, and random numbers were generated using SPSS 25.0 (SPSS Inc., Chicago, IL, USA) statistical software. This study is single-blinded. To minimize evaluation bias, assessors responsible for screening and outcome assessment will be blinded to group assignments. The anesthesiologist will be aware of each patient's group but will remain isolated from the research results. Patients will not be informed of their group allocation. Randomization will be conducted using a computer-generated blocked randomization sequence. A nurse will generate the allocation sequence and prepare sealed, numbered envelopes. The anesthesiologist will open each envelope only when the patient enters the operating room.

### 2.6 Statistical analysis

SPSS 25.0 (SPSS Inc., Chicago, IL, USA) statistical software was used for data analysis. Measurement data were expressed as mean ± standard deviation (SD), and non-normally distributed data were expressed as median [interquartile range] [Median, IQR (Q25–Q75)]. The independent sample t-test was applied to compare normally distributed data between groups. Repeated measures analysis of variance was used to compare normally distributed data across different time points between the two groups, with the Bonferroni test applied for pairwise comparisons at each time point. For non-normally distributed data, a generalized estimating equation (GEE) model was used to compare differences in relevant indicators between the two groups at different time points and to clarify the interaction between time and grouping. Cross-group comparisons of adverse event incidence were conducted using the Chi-square (*χ*^2^) test. The *p* value of <0.05 was considered statistically significant.

### 2.7 Sample size evaluation

The PASS 15.0 software package was used to determine the sample size. The significance level (*α*) was set at 0.05, and the power (*β*) at 0.2. The reduction in VAS score at 24 h post-surgery was chosen as the primary outcome. Based on previous studies and reports, the difference in VAS score reduction between the SFNB and CFNB groups at 24 h post-surgery was −0.5. The non-inferiority margin (*δ*) was set at 1, and a non-inferiority test was applied. The sample size was calculated to be equal for both the SFNB and CFNB groups, with a SD of 0.9 and 0.7, respectively. The required sample size was determined to be 42. Accounting for a follow-up loss rate of less than 10%, the final sample size was set at 50 per group, with a total of 100 subjects.

## 3 Results

### 3.1 Basic information

**Figure 1:**
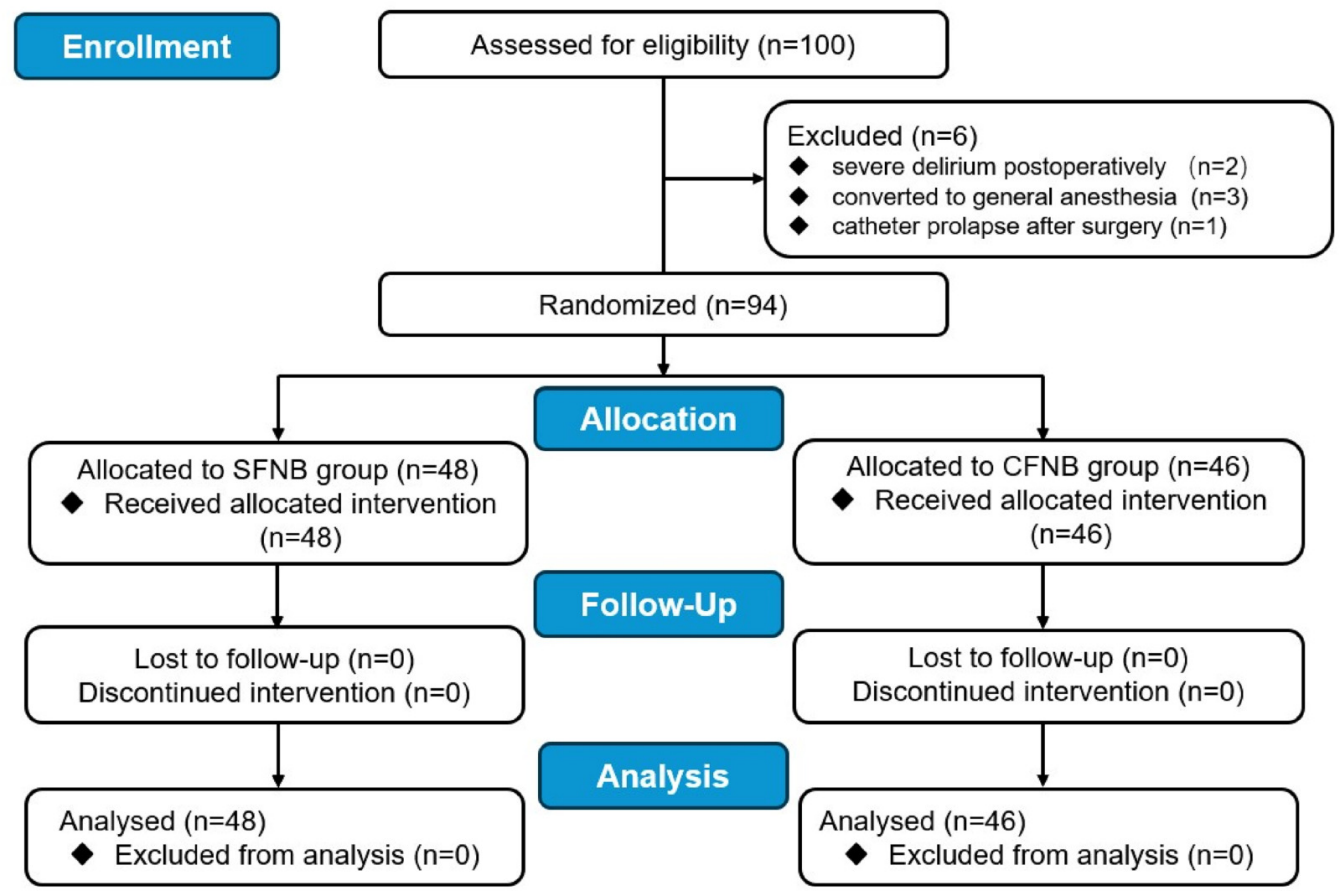
Flow diagram of this study. SFNB, single femoral nerve block; CFNB, Continuous femoral nerve block; TKA, total knee arthroplasty.

A total of 94 patients successfully completed the study. In the SFNB group, 1 patient developed severe delirium postoperatively, and 1 case was converted to general anesthesia due to spinal anesthesia failure. Both were excluded, leaving 48 patients who completed the study. In the CFNB group, 2 patients required general anesthesia, 1 patient developed delirium, and 1 patient experienced catheter prolapse on the first postoperative day. These patients were also excluded, leaving 46 patients for final analysis (Figure 1).

There were no statistically significant differences between the two groups in terms of age, gender distribution, body mass index, ASA grading, previous medical history (cardiovascular disease, cerebrovascular disease, pulmonary disease, diabetes, smoking history), left or right knee surgery, or surgery time (*p* > 0.05).

Besides, the two groups were comparable with no significant differences in surgery limb, preoperative knee VAS score, AKS knee score and function score, MMT score, as well as ROM (*p* > 0.05), as shown in Table 1.

**Table 1:** Demographic and surgical variables (mean ± SD).

Indexes	SFNB	CFNB	*t*/*X^2^* value	*P* value
Number	48	46	—	—
Demographic variables				
BMI (kg/m^2^)	24.6 ± 1.5	24.2 ± 1.4	1.378	0.171
Gender Male/Female	28/20	22/24	1.042	0.307
Age	82.6 ± 8.3	83.1 ± 6.8	0.349	0.728
Dementia, *n* (%)	19 (39.6)	15 (32.6)	0.495	0.482
Cardiovascular disease, *n* (%)	15 (31.3)	16 (34.8)	0.133	0.716
Neurological disease, *n* (%)	15 (31.3)	11 (23.9)	0.632	0.427
Pulmonary disease, *n* (%)	4 (8.3)	5 (10.9)	0.175	0.676
Diabetes, *n* (%)	16 (33.3)	19 (41.3)	0.639	0.424
Smoking (Yes/No)	23 (47.9)	20 (43.5)	0.186	0.666
ASA score II/III	14 (29.2)	11 (23.9)	0.332	0.564
Surgical variables				
Surgery time (min)	117.0 ± 19.3	117.8 ± 21.1	0.198	0.843
Surgery limb (Left/Right)	27 (56.3)	23 (50.0)	0.369	0.544
Preoperative knee VAS score	5[4,5]	5[4,6]	0.394	0.394
Preoperative AKS knee score	32.1 ± 6.9	33.5 ± 7.5	0.912	0.364
Preoperative AKS function score	42.7 ± 4.5	41.7 ± 4.2	1.208	0.230
Preoperative MMT score	4.6 ± 0.5	4.7 ± 0.5	1.333	0.186
Preoperative ROM (◦)	60.7 ± 7.6	61.8 ± 7.6	0.713	0.478

TKA, total knee arthroplasty; SFNB, single femoral nerve block; CFNB, continuous femoral nerve block; BMI, body mass index; VAS, visual analog score; ROM, range of motion; MMT, manual muscle test; AKS, American knee society knee score.

### 3.2 Comparison of postoperative VAS at different time points

The VAS was used to evaluate pain at rest and during movement at different time points. The results indicated that both SFNB and CFNB provided effective postoperative analgesia. There were no significant differences in VAS scores for both resting and activity-related pain between the two groups at 2 h, 6 h, 12 h, 24 h, 48 h, and 72 h postoperatively (*p* > 0.05). Within each group, the results showed that the analgesic effect of both SFNB and CFNB lasted for 72 h post-surgery. Additionally, there was no significant difference in the total dose of celecoxib taken between the two groups after surgery (*p* > 0.05). Considering the interaction between time and groups on the results, we used the generalized estimating equation (GEE) model to further compare differences in VAS (rest pain and motion pain) between the two groups at different time points. The effect significance tests showed that the interaction between time and groups on VAS (rest pain) and VAS (motion pain) were not statistically significant (Wald Chi-Square _rest pain_ = 1.369, *P*_rest pain_ = 0.928 > 0.05; Wald Chi-Square _motion pain_ = 0.466, *P*_motion pain_ = 0.993 > 0.05), which proved that there were no significant interaction on the VAS results between time and groups, as shown in Table 2.

**Table 2:** Comparison of postoperative VAS at different time points (median, IQR).

Indexs	Time	SFNB	CFNB	*Z* value	*P* value
VAS (rest pain)	2 h after surgery	1 [0, 1]	1 [0, 1]	0.107	0.915
	6 h after surgery	1 [0, 1]	0.5 [0, 1]	0.310	0.756
	12 h after surgery	1 [1, 1.75]	1 [0, 2]	0.699	0.484
	24 h after surgery	2 [1.25, 3]	2 [1, 2]	0.516	0.606
	48 h after surgery	2 [1, 2]	1 [1, 2]	0.543	0.587
	72 h after surgery	1 [1, 2]	1 [1,2]	1.119	0.263
VAS (motion pain)	2 h after surgery	2 [1, 2]	2 [0.75, 2]	0.773	0.439
	6 h after surgery	2 [1, 2]	1.5 [1, 3]	0.267	0.789
	12 h after surgery	2 [1.25, 3]	2 [1, 2]	1.206	0.228
	24 h after surgery	3 [2, 3]	2.5 [2, 3]	0.668	0.504
	48 h after surgery	2 [1, 3]	2 [1, 3]	0.809	0.419
	72 h after surgery	2 [1, 3]	2 [1, 3]	1.110	0.267
Celecoxib (mg)	—	0 [0, 200]	0 [0, 200]	0.484	0.628

TKA, total knee arthroplasty; SFNB, single femoral nerve block; CFNB, continuous femoral nerve block; VAS, visual analog score.

### 3.3 Comparison of quadriceps muscle strength and knee function

The results demonstrated that quadriceps muscle strength in both groups began to recover gradually starting 2 h after surgery. Between-group comparisons showed that the MMT scores in the SFNB group were significantly higher than those in the CFNB group (*p* < 0.05) at 6 h, 12 h, and 24 h post-surgery. Similarly, the knee ROM in the SFNB group was significantly better than in the CFNB group at 24 h post-surgery. There were no significant differences in muscle strength between the two groups 2 days postoperatively (*p* > 0.05), nor were there significant differences in knee ROM and AKS scores at 3 days (*p* > 0.05) and 1 week after surgery (*p* > 0.05). We also used the GEE model to compare differences in MMT score between the two groups at different time points. The effect significance test showed that there were significant differences between multiple time measurements (Wald Chi-Square_MMT score_ = 18.759, *P*_MMT score_ = 0.002 < 0.05). We further compared the difference of MMT score at six times by correcting the interference of time factors. The results showed that there were no significant differences on the MMT score between the two groups at the time of 2 h, 48 h and 72 h after surgery (*P*_2h after surgery_ = 0.072 > 0.05; *P*_48h after surgery_ = 0.371 > 0.05; *P*_72h after surgery_ = 0.535 > 0.05); while the MTT score of the SFNB group were all significantly higher than those of the CFNB group at the other three times. (*P*_6h after surgery_ = 0.001 < 0.05; *P*_12h after surgery_ = 0.003 < 0.05; *P*_24h after surgery_ = 0.001 < 0.05), as shown in Table 3.

**Table 3:** Comparison of quadriceps muscle strength and knee function (mean ± SD).

Indexs	Time	SFNB	CFNB	*t* value	*P* value
Postoperative MMT score	2 h after surgery	1.46 ± 0.74	1.20 ± 0.69	1.778	0.079
	6 h after surgery	2.85 ± 0.74	2.37 ± 0.74	3.164	0.002
	12 h after surgery	3.81 ± 0.61	3.37 ± 0.85	2.911	0.005
	24 h after surgery	4.69 ± 0.45	4.30 ± 0.63	3.363	0.001
	48 h after surgery	4.96 ± 0.21	4.91 ± 0.28	0.892	0.375
	72 h after surgery	4.99 ± 0.14	4.96 ± 0.21	0.619	0.537
Postoperative ROM (°)	24 h after surgery	44.48 ± 11.08	38.83 ± 11.93	2.382	0.019
	72 h after surgery	69.44 ± 10.17	65.65 ± 12.11	1.644	0.104
	1 w after surgery	93.63 ± 10.68	90.48 ± 10.41	1.446	0.152
AKS knee score	1 w after surgery	87.02 ± 7.61	84.61 ± 7.99	1.498	0.138
AKS function score	1 w after surgery	86.83 ± 6.60	85.17 ± 7.13	1.172	0.244

TKA, total knee arthroplasty; SFNB, single femoral nerve block; CFNB, continuous femoral nerve block; BMI, body mass index; ROM, range of motion; MMT, manual muscle test; AKS, American knee society knee score.

### 3.4 Comparison of complications during hospitalization

The complications contained four aspects, including puncture complications, circulatory complications, inflammation, central system complications, and so on. The results showed that, in the SFNB group, 3 patients experienced nausea and vomiting, 1 had vertigo, 2 developed bradycardia, 3 had fevers, and 1 patient had a pulmonary infection; all complications were resolved successfully. In the CFNB group, nausea and vomiting occurred in 2 patients, bradycardia in 1 patient, and fever in 2 patients. There were no significant differences in complications or LOS between the two groups (*p* > 0.05), as shown in Table 4.

**Table 4:** Comparison of related complications between the two groups [*n*, (%)].

Indexes	SFNB	CFNB	*t*/*X^2^* value	*P* value
*n*	48	46	—	—
Puncture complications				
Local hematoma	0 (0)	0 (0)	—	—
Postoperative sensory disturbance	0 (0)	0 (0)	—	—
Postoperative dyskinesia	0 (0)	0 (0)	—	—
Circulatory complications				
Bradycardia	2 (4.17)	1 (2.17)	0.308	0.579
Hypotension	0 (0)	0 (0)	—	—
Hypertension	0 (0)	0 (0)	—	—
Cardiac insufficiency	0 (0)	0 (0)	—	—
Arrhythmia	0 (0)	0 (0)	—	—
Central system complications				
Nausea and vomiting	3 (6.25)	2 (4.35)	0.170	0.681
Headache	0 (0)	0 (0)	—	—
Dizziness	0 (0)	0 (0)	—	—
Inflammation				
Fever	3 (6.25)	2 (4.35)	0.170	0.681
Urinary infection	0 (0)	0 (0)	—	—
Pulmonary infection	1 (2.08)	0 (0)	1.354	0.244
Surgical incision infection	0 (0)	0 (0)	—	—
Deep venous thrombosis	0 (0)	0 (0)	—	—
Others				
Urinary retention	0 (0)	0 (0)	—	—
Pruritus	1 (2.08)	0 (0)	1.354	0.244
LOS (length of stay)	5.65 ± 1.27	5.96 ± 1.05	1.276	0.205

TKA, total knee arthroplasty; SFNB, single femoral nerve block; CFNB, continuous femoral nerve block; BMI, body mass index; ROM, range of motion; MMT, manual muscle test; AKS, American knee society knee score.

### 3.5 Comparison of the GCQ scores in the two groups

As shown in Table 5, the physiological and psychological assessment scores of the GCQ in the SFNB group were significantly higher than those in the CFNB group (*p* < 0.05). However, there were no significant differences in the social-cultural and environmental scores between the two groups (*p* > 0.05). In the CFNB group, two-thirds of the patients reported psychological and physical discomfort during daily activities or rehabilitation exercises, due to the presence of the catheter, seepage from the PCA, and pruritus caused by the adhesive tape.

**Table 5:** Comparison of the GCQ scores between the two groups (mean ± SD).

Score	SFNB	CFNB	*t* value	*P* value
*n*	48	46	—	—
Physiological	3.50 ± 0.65	3.13 ± 0.69	2.676	0.009
Psychological	3.15 ± 0.74	2.78 ± 0.78	2.302	0.024
Social-cultural	3.29 ± 0.71	3.17 ± 0.68	0.820	0.414
Environmental	3.17 ± 0.69	2.96 ± 0.82	1.347	0.181

SFNB, single femoral nerve block; CFNB, continuous femoral nerve block.

## 4 Discussion

In recent years, the incidence of knee joint diseases has risen, and TKA surgery has been widely adopted (10). Knee replacement is commonly used to alleviate pain caused by severe knee function degradation, correct deformities, and improve quality of life (11). However, postoperative pain often hinders early joint rehabilitation, slowing knee recovery. This highlights the need for an effective analgesic method for such surgeries.

Patient-controlled intravenous analgesia (PCIA) is frequently used in clinical practice; however, it can cause sedation along with pain relief (12). Given that most TKA patients are elderly, sedation may reduce alertness, impeding early postoperative training (13). Research has shown that the femoral nerve primarily controls sensation in the anterior thigh and the knee through its branches (14). Therefore, ultrasound-guided femoral nerve block (FNB) can be an effective multimodal analgesic approach (15). CFNB provides strong analgesia and is considered the “gold standard” for postoperative pain management in TKA patients (16, 17). However, CFNB can impair muscle strength and delay early postoperative training. Additionally, femoral nerve catheterization can reduce patient comfort (16, 18). This study aimed to explore the analgesic effects of SFNB using 0.2% ropivacaine (50 ml) to offer a reference for improved clinical pain management strategies.

In this study, we found that compared with traditional CFNB, there were no statistically significant differences in VAS scores at rest and during activity at 2 h, 6 h, 12 h, 24 h, 48 h, and 72 h after surgery, nor in the dosage of postoperative celecoxib used for remedial analgesia. These results suggest that SFNB can provide adequate analgesia lasting up to 72 h postoperatively (19). In addition to quadriceps muscle strength, we also examined the ROM and the AKS score. Muscle strength was observed to recover gradually starting 2 h after TKA, and knee joint mobility resumed within 6 h post-surgery. However, the MMT grade and ROM in the SFNB group were significantly higher than in the CFNB group at 6 h, 12 h, and 24 h after surgery. We also used GEE model to further prove the differences in VAS (rest pain and motion pain) and MTT score between the two groups at different time points. The results obtained by GEE model were all similar with those by Rank sum test and independent sample *t* test. All the findings indicate that SFNB not only provides stable and effective analgesia but also significantly promotes the recovery of postoperative muscle strength and joint mobility, supporting overall postoperative rehabilitation (20). By 3 days and 1-week post-surgery, there was no significant difference in AKS scores between the two groups, suggesting that patients in both groups experienced a steady recovery as the drugs were metabolized and maintained consistent efficacy.

In clinical, there are many factors, including the type, concentration and volume of local anesthetics. Moreover, the relationship between the perineuronal spatial anatomy and the target nerve/plexus may have a decisive influence on the effect of local anesthetics. The femoral nerve space is a large space, and the volume of local anesthetics has become an important factor affecting the anesthetic effect. Thus, compared with 0.2% ropivacaine (20 ml), 0.2% ropivacaine (50 ml) had a wider block plane, and could fully infiltrate the femoral nerve, with a longer total sensory block time.

Regarding complications, we discussed the puncture complications, circulatory complications, inflammation, central system complications, and so on. Pre-existing comorbidities significantly impact postoperative outcomes. Cardiovascular/cerebrovascular diseases may induce severe hemodynamic fluctuations. Pulmonary comorbidities and smoking history could predispose patients to pulmonary infections and pyrexia. Dementia and cerebrovascular disorders may lead to postoperative communication difficulties. Diabetes mellitus could increase risks of infections and postoperative pain. Thus, we firstly compared the dementia, cardiovascular disease, neurological disease, pulmonary disease, diabetes, and smoking between the two groups. There were no statistically significant difference, which implied that the two groups had comparable baseline characteristics, including physical status and comorbidities.

According to the postoperative complications, We found that: (1) there were no puncture complications, such as local hematoma, postoperative sensory disturbance and dyskinesia, which implied the SFNB and CFNB were safe. (2) According to the circulatory complications. there were two cases of bradycardia in SFNB group and one case of bradycardia in CFNB group, which had nothing to do with the FNB. We thought the bradycardia was related to the age and patient's basal heart rate. (3) Postoperative nausea and vomiting were relatively common in the two groups, which may be linked with the circulatory fluctuations or stimulation of vestibular function induced by moving the patient and postural changes. (4) Fever was also relatively common, which maybe caused by the weak resistance of the elderly patients and the operation stimulation. (5) Besides, femoral nerve block could facilitate early postoperative active movement and relieve pain for patients. However, continuous femoral nerve block, due to the presence of the catheter, could cause some discomfort during active training.

The results indicated that SFNB with a low concentration and high volume of anesthetic is safe and suitable for widespread clinical use. Although the difference in hospital stay between the two groups was not statistically significant, the average length of stay in the SFNB group was slightly shorter than in the CFNB group, suggesting that early postoperative training might help accelerate rehabilitation, which need to be further discussed.

Additionally, the General Comfort Questionnaire (GCQ) (21, 22) was used to evaluate patient comfort 72 h after surgery. We found that both the physiological and psychological assessment scores in the SFNB group were significantly higher than those in the CFNB group. Two-thirds of the patients in the CFNB group reported feeling psychological and physical discomfort during daily activities and rehabilitation exercises due to the presence of the catheter, seepage from the PCA, and pruritus caused by the adhesive tape (23, 24). These findings suggest that continuous femoral nerve block may increase patient discomfort after surgery. In contrast, single femoral nerve block not only provides effective pain relief and supports postoperative functional training but also improves overall patient comfort.

However, this study has certain limitations: (1) The study was limited to two groups comparing the analgesic effects of SFNB and CFNB combined with PCIA. The minimum effective concentration of ropivacaine for this procedure in elderly patients needs further investigation. (2) This study was conducted at a single center with a relatively small sample size. Future multicenter randomized controlled trials with larger sample sizes are necessary to provide more robust evidence for pain management strategies.

## 5 Conclusion

SFNB with 0.2% ropivacaine (50 ml) offers effective and stable postoperative analgesia that is both safe and straightforward. Using low-concentration, high-volume ropivacaine can accelerate the recovery of muscle strength following TKA, facilitating early postoperative training and promoting faster recovery.

## Data Availability

The original contributions presented in the study are included in the article/Supplementary Material, further inquiries can be directed to the corresponding authors.
